# Political economy analysis of subnational health management in Kenya, Malawi and Uganda

**DOI:** 10.1093/heapol/czad021

**Published:** 2023-04-11

**Authors:** Daniela C Rodríguez, Lakshmi Narasimhan Balaji, Elita Chamdimba, Juba Kafumba, Adam D Koon, Jacob Mazalale, Dadirai Mkombe, Joshua Munywoki, Tawonga Mwase-Vuma, Justine Namakula, Bejoy Nambiar, Abigail H Neel, Xavier Nsabagasani, Ligia Paina, Braeden Rogers, Maxton Tsoka, Evelyn Waweru, Alister Munthali, Freddie Ssengooba, Benjamin Tsofa

**Affiliations:** International Health, Johns Hopkins Bloomberg School of Public Health, 615 North Wolfe Street, Baltimore, MD 21205, USA; Health Programme, UNICEF New York, 3 UN Plaza, New York, NY 10017, USA; Centre for Social Research, University of Malawi, P.O. Box 280, Zomba, Malawi; Centre for Social Research, University of Malawi, P.O. Box 280, Zomba, Malawi; International Health, Johns Hopkins Bloomberg School of Public Health, 615 North Wolfe Street, Baltimore, MD 21205, USA; Centre for Social Research, University of Malawi, P.O. Box 280, Zomba, Malawi; Centre for Social Research, University of Malawi, P.O. Box 280, Zomba, Malawi; KEMRI-Wellcome Trust Research Programme, Hospital Road, P.O. Box 230, Kilifi, Kenya; Centre for Social Research, University of Malawi, P.O. Box 280, Zomba, Malawi; School of Public Health, Makerere University, New Mulago Hill Road, Mulago, Kampala, Uganda; UNICEF Malawi, PO Box 30375, Airtel Complex Area 40/31, Lilongwe, Malawi; International Health, Johns Hopkins Bloomberg School of Public Health, 615 North Wolfe Street, Baltimore, MD 21205, USA; School of Public Health, Makerere University, New Mulago Hill Road, Mulago, Kampala, Uganda; International Health, Johns Hopkins Bloomberg School of Public Health, 615 North Wolfe Street, Baltimore, MD 21205, USA; Health Section, UNICEF Eastern and Southern Africa Regional Office, United Nations Complex, Gigiri, P.O. Box 44145-00100, Nairobi, Kenya; Centre for Social Research, University of Malawi, P.O. Box 280, Zomba, Malawi; KEMRI-Wellcome Trust Research Programme, Hospital Road, P.O. Box 230, Kilifi, Kenya; Centre for Social Research, University of Malawi, P.O. Box 280, Zomba, Malawi; School of Public Health, Makerere University, New Mulago Hill Road, Mulago, Kampala, Uganda; KEMRI-Wellcome Trust Research Programme, Hospital Road, P.O. Box 230, Kilifi, Kenya

**Keywords:** Political economy, decentralization, health systems, management, governance, Kenya, Malawi, Uganda

## Abstract

The need to bolster primary health care (PHC) to achieve the Sustainable Development Goal (SDG) targets for health is well recognized. In Eastern and Southern Africa, where governments have progressively decentralized health decision-making, health management is critical to PHC performance. While investments in health management capacity are important, so is improving the environment in which managers operate. Governance arrangements, management systems and power dynamics of actors can have a significant influence on health managers’ ability to improve PHC access and quality. We conducted a problem-driven political economy analysis (PEA) in Kenya, Malawi and Uganda to explore local decision-making environments and how they affect management and governance practices for health. This PEA used document review and key informant interviews (*N *= 112) with government actors, development partners and civil societies in three districts or counties in each country (*N* = 9). We found that while decentralization should improve PHC by supporting better decisions in line with local priorities from community input, it has been accompanied by thick bureaucracy, path-dependent and underfunded budgets that result in trade-offs and unfulfilled plans, management support systems that are less aligned to local priorities, weak accountability between local government and development partners, uneven community engagement and insufficient public administration capacity to negotiate these challenges. Emergent findings suggest that coronavirus disease 2019 (COVID-19) not only resulted in greater pressures on health teams and budgets but also improved relations with central government related to better communication and flexible funding, offering some lessons. Without addressing the disconnection between the vision for decentralization and the reality of health managers mired in unhelpful processes and politics, delivering on PHC and universal health coverage goals and the SDG agenda will remain out of reach.

Key messages

- Decentralization not only can bring decision-making closer to local governments and communities but also leads to additional bureaucratic layers that can constrain the discretion of managers who face difficult trade-offs between comprehensive but idealistic health plans that cannot be implemented with available budget envelopes.
- Health plans and budgets are misaligned due to chronic underfunding and lack of major investments in health systems leading to frustration and mistrust in the planning process.
- Shifting power dynamics within the health system and within districts lead to unclear roles and responsibilities amid a growing number of actors involved in health management and governance.
- Accountability mechanisms linked to decentralization create important demands and expectations for community engagement in health planning, but these processes can be time-consuming with high transaction costs leading to uneven adherence.
- Power dynamics, underfunded plans and weak accountability structures are complicated by the presence of influential development partners at national and levels.

## Introduction

In 2018, the global community reconfirmed primary health care (PHC) as the bedrock of efforts to achieve universal health coverage (UHC) and Sustainable Development Goal (SDG) 3 (WHO & UNICEF, 2018a). PHC aims to ensure high-quality essential health services and public health functions that are close to where people live, supported by empowered communities and bolstered by multisectoral action to address social determinants of health (WHO & UNICEF, 2018b). Despite wide rhetorical support for this ambition, in reality, most countries in Eastern and Southern Africa (ESA) require significantly increased financial investments and reforms to ensure that health systems are able to effectively deliver universal access to high-quality PHC, a critical component to achieving UHC (de Maeseneer *et al.*, 2020). In 2021, 19 of 21 ESA countries were off-track for achieving the under-five mortality target by 2030 (United Nations Inter-Agency Group For Child Mortality Estimation (UN IGME), 2021), and all but one country were below the global average on the UHC Service Coverage Index (SDG target 3.8.1) [World Health Organization (WHO)], a composite indicator measuring coverage of essential health services through 14 tracer indicators.

Effective health sector decentralization is integral to achieving PHC and UHC. Decentralization refers to the process of transferring political, fiscal or administrative authority from central agencies, like Ministries of Health (MoHs), to actors, such as county and district health management teams (DHMTs), with the goal of improving efficiency, quality and equity in health service delivery (Bossert, 2002). Decentralization can take many forms, including ‘deconcentration’ (authority moves to regional institutions within MoH), ‘devolution’ (authority moves to governments) or ‘delegation’ (parastatals are given new powers) (Rondinelli, 1981).

As countries progressively decentralize, health management teams are meant to assume increasing responsibility and leadership for planning, budgeting and delivery of PHC services, yet by-and-large there have not been concomitant investments in strengthening governance, leadership and management at local levels. Both technical and political actors share the responsibility of developing annual implementation plans that draw from national health plans. Developing these plans should ideally be participatory, drawing on community engagement and bottom-up activities and priorities, which are used to allocate funding that units receive from the national level. However, in practice, guidelines for health planning and budgeting are often not followed and annual plans are not fully implemented. Bossert and others have studied the impact of decentralization on the range of choices available to managers as well as their actual ability to take decisions that are nominally within their authority, also known as ‘decision space’ (Bossert, 1998; 2002; Bossert and Mitchell, 2011). Decision space varies by decentralized function with trade-offs occurring within and across functions and can lead to local decision-making not necessarily aligning with national priorities (i.e. principal–agent problem) (Bossert, 2002). Challenges in coordination and collaboration between national and levels are common, and -level actors often cannot exercise the authority and decision-making responsibilities they are assigned (Rondinelli, 1981; Bossert, 1998).

Recognizing this tension around effective management, the WHO published a Leadership and Management Strengthening Framework as part of its ‘Making Health Systems Work’ series in 2007 (Egger and Ollier, 2007). This framework calls for investments not only in building professional management cadres for the health sector (numbers of managers) and developing management competency (knowledge and skills) but also in management support systems (e.g. systems and processes to support management functions for planning, budgeting and personnel management) and improvements in the enabling environment (e.g. decision space, accountability structures and incentive systems), which are often mired in politics and mostly neglected in health systems strengthening programmes.

Research on health governance, leadership and management in low- and middle-income countries (LMICs) has had limited engagement with politics (de Leeuw *et al.*, 2014), particularly as it relates to UHC (Fox and Reich, 2015; Sparkes *et al.*, 2019; Rizvi *et al.*, 2020). This is surprising since moving towards greater emphasis on PHC and UHC involves both national and actors making difficult, often political choices about the distribution of public goods and services (WHO, 2010; Hanson *et al.*, 2022). The choices, and the forms of deliberation that lead to them, reflect deeper social values that get institutionalized over time (Koon *et al.*, 2020), formal and informal arrangements characterized by changing rules of the game, as well as power dynamics between global financing organizations and national and actors. Scholars of health systems strengthening in LMICs often argue that ‘political will’ or ‘political commitment’ is a necessary precondition for improving PHC and achieving UHC (Nicholson *et al.*, 2015; Yamey and Evans, 2015). This characterization, however, is insufficient and has led to calls for research that better accounts for complex social processes in advancing health goals (Jawad, 2019; Reich, 2019).

### Study approach

Recently, scholars have turned to political economy analysis (PEA) as a means of providing explanations for the interaction of interests, institutions and ideas that determine health systems function or reform (Rizvi *et al.*, 2020; Sparkes *et al.*, 2022). PEA is an analytical tool useful for understanding the incentives, power dynamics, interests, behaviours and constraints in a system and how these influence resource distribution, policy and programmatic decisions over time. Drivers of political economy are centred on structural issues, coalition of interests, institutional dynamics and stakeholder relationships (UK-DFID, 2009; Harris, 2013; Fritz, 2014). In the field of overseas development assistance, it also captures the role that global-level processes and actors play on national-level decision-making.

Problem-driven PEA focuses on a specific problem or policy, as opposed to a whole country or sector: to better understand a challenging issue, the institutional dynamics is contributing to the problem, and the broader actors and systems factors that facilitate or hinder change (UK-DFID, 2009; Harris, 2013; Fritz, 2014). A critical feature of problem-oriented PEA is its operational, practice-oriented nature, which generates more practicable, politically realistic recommendations that consider the risks of taking action in a particular context (UK-DFID, 2009; Harris, 2013; Fritz, 2014).

As part of a United Nations Children’s Fund (UNICEF)-supported initiative aimed to strengthen PHC management in Kenya, Malawi and Uganda, we undertook a PEA to better characterize the complex environments in which health managers operate, including the factors that enable and inhibit good health systems management practices. To this end, our PEA examined the health systems context, actor dynamics, policy environment, local authorities and constituent communities to understand power relations, governance arrangements and accountability structures that influence decision-making, including between local authorities and communities. Furthermore, the PEA takes a broader view of governance and management with the goal of identifying potential leverage points for future activities.

Results from individual study countries are available in country-specific publications (Munthali *et al.*, 2021; Ssengooba *et al.*, 2021; Tsofa *et al.*, 2023) led by the Kenya, Malawi and Uganda teams. In this article, we present cross-country findings—specifically, persistent challenges to health governance and management found across all three settings, and recommendations to address these.

## Materials and methods

### Study design

We pursued an embedded multiple case study design with three counties/districts in each country as the embedded unit of analysis resulting in one overarching case per country followed by cross-case analysis (Yin, 2009). The study used qualitative methodology, including document review and key informant interviews (KIIs). To the extent that financial and spending data were available, quantitative analysis of trends in allocation and expenditure was also conducted.

**Figure 1.**
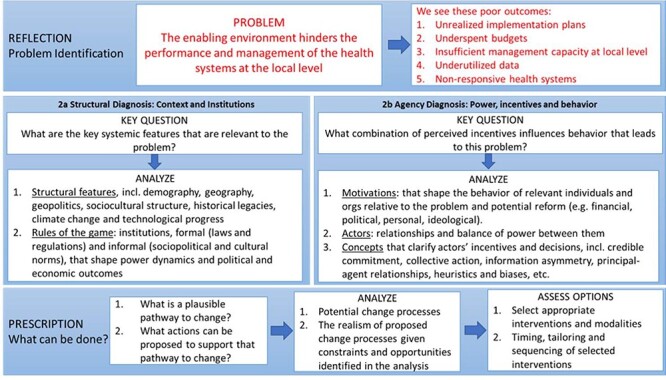
PEA theoretical framework, adapted from Siddiqi *et al.*, (2009)

Figure 1 illustrates the analytical pathway for the problem-driven PEA, as adapted from Siddiqi *et al*. (2009), that we used to guide this study. The framework helps make explicit the problem and the poor outcomes to which it leads, which is then followed by two types of diagnoses. The structural diagnosis is focused on how the context and the rules of the game, such as formal and informal norms, influence the problem and its outcomes. The agency diagnosis concerns itself with the actors involved, power dynamics between them and what incentives or unconscious biases motivate their behaviours. Given the resulting diagnoses, analysts then identify plausible pathways for change.

**Table 1.** Health systems and policy context in PEA study countries

	Kenya	Malawi	Uganda
Country population (2020)	53.77M	19.13M	45.74M
Number of districts/counties	47	28	136
Current health expenditure per capita (USD) (2019)^b^	$83	$30	$32
Domestic general government health expenditure (% of current health expenditure) (2019)^b^	46 Since 2014/15, increase in budgetary allocation in two of the three study counties; erratic in one county	33 Government spending on health in study districts has generally declined since 2015/16	15 Negligible increase in government allocations to study districts between 2018/19 and 2020/21
External health expenditure (% of current health expenditure) (2019)^b^	19	44	42
Under-five mortality rate (deaths per 1000 live births) (2020)^c^	42	39	43
National UHC agenda	National UHC rollout in 2018 as part of Government Big 4 Agenda	Essential health package (EHP) adopted in 2004, continually revised. Most recent Health Sector Strategic Plan II (2017–22) aims to achieve UHC via the EHP	Goal of the Health Sector Development Plan II (2015/16–2019/20) is to accelerate achievement of UHC
Key characteristics of decentralization for health management	47 autonomous counties established in 2013 following devolution. Substantial expansion of actors in health management	District councils received mandate health management in 2005, but shift of responsibility happened functionally in 2019–20	Decentralization started in 1997, leading to significant proliferation of districts from 45 to 136 in 2021
Examples of decentralization domains that have not been implemented	Conditional grants from national levels to counties targeting specific health systems strengthening interventions HRH production and in-services training Health sector regulatory functions	Drug procurement Capital budget Human resource management	Policy formulation and planning
Existing mechanisms for interacting with donors	Yes	Yes	Yes

a Worldometer. https://www.worldometers.info/world-population.

b WHO Global Health Expenditure Database https://apps.who.int/nha/database/Select/Indicators/en.

c UNICEF 2021 Child Mortality Report: https://childmortality.org/wp-content/uploads/2021/12/UNICEF-2021-Child-Mortality-Report.pdf.

For the purposes of this study, the problem is defined as ‘The enabling environment hinders the performance and management of the health systems at the local level’ which leads to a series of poor performance outcomes. The study was organized according to this analytical frame, and we interrogated each of the core components of this framework (e.g. structural diagnosis, agency diagnosis and pathways for change) through common data collection instruments and data analysis approach.

### Study context

The relational dynamics driving health governance and management hinge, in part, on the structures and institutions that have developed over the last several decades. Table 1 provides a snapshot of the health systems and policy context in our three study countries, including health expenditures and policy history for UHC.

In this study, decentralization is a defining characteristic of each of the health systems. The degree and manner of implementation vary between contexts (see Box 1). For example, in Kenya, the swift pace at which devolution was implemented preceded the build-up of county-level structures and managerial capacity to manage its new functions, including health workforce and commodity supplies, leading to disruptions in the health system that took time to resolve (Tsofa *et al.*, 2017a). For example, greater autonomy on human resources for health (HRH) decisions at county level led to politicization of staff recruitment and deployment (Tsofa *et al.*, 2017a).

Box 1. Decentralization context of three study countries

**KENYA**

Although the current 47 semi-autonomous counties were established in 2013 following ratification of a new constitution in 2010 and subsequent devolution, health sector decentralization reform has been ongoing since the 1980s, leading to an expansion of the number of actors involved in health management and growing autonomy at the county level over resource allocations. Greater public and community engagement in health planning and budgeting has resulted alongside increasing complexity given overlapping roles between actors and challenges in balancing technical and political priorities. Kenya is the only study country where health financing has increased in the past several years. Nonetheless, erratic and late disbursement of county funds from the National Treasury continues to affect implementation (Tsofa *et al*., 2021).

**MALAWI**

District councils (DCs) received a mandate to lead planning, budgeting and delivery of primary and secondary health services in 2005, following the enactment of the Local Government Act in 1998. The Director of Health and Social Services (DHSS) role, which had been established in 2005 and forms part of the DC, was functionally filled in each district in 2019–20, with the DHMT reporting to the DHSS. While DCs are tasked with implementing—and not deviating—from the district implementation plan, in reality the central level retains some political influence over health decisions, and the DHSS sometimes bypasses DCs by taking technical issues directly to the Ministry of Health and Population. Since decentralization, districts are now able to make resource allocations themselves and are in control of hiring lower-level staff; however, the central government retains control over specific elements of the health system, such as drug procurement, capital budgets and recruitment of higher-ranking officials. Malawi has ongoing issues related to scarce resources, partly resulting from a historical budget ceiling that limits health spending. The central government has control over district-level budget allocations and disbursement of funds is unpredictable, often falling short of planned budgets (Munthali *et al.*, 2021).

**UGANDA**

Decentralization was initiated in 1997 with the aim to devolve service delivery responsibilities to subnational governments. There have been significant and ongoing amendments to this framework in the decades since, including tripling the number of districts (e.g. 45 in 1997 to 136 by 2021). Uganda has instituted several innovative health sector reforms including the introduction of several digital solutions, such as the Integrated Financial Management System, fiscal transfer systems and a Personnel and Payroll System; decentralizing wage and payroll management and upgrading the budget formulation and implementation processes. However, the rate of these reforms has been rapid, and at times, district capacity development has not kept pace with the introduction of technological innovations. Scholars have noted the devolution of responsibilities was not accompanied by a commensurate increase in resources, nor do district governments generate local revenues (as of 2005) (Ssengooba *et al.*, 2021).

Under relatively new devolution arrangements in Malawi, remnants of MoH-centralized control of all health sector activities still hold sway and limit local decision-making (i.e. hierarchical power). Even though decision-making authority is allocated to District Councils on paper, district health officers can bypass Councils by directly working with and reporting to central MoH (Bulthuis *et al.*, 2021).

After decentralization, districts in Uganda spent more on curative care than primary care which led to conditional health grants meant to encourage investment in PHC (Bossert, 2002). Furthermore, while substantial power is connected to hiring, firing and supervision of health personnel, large salary obligations for HRH mean substantially less discretionary resources for other line items in district budgets (Bossert, 2002). In addition, the District Health Committee still occupies more decision space than the DHMT even though hierarchical power is less prominent than before (Bulthuis *et al.*, 2021).

Decentralization has also had financial implications for district health team operations and health financing in each setting. In all three settings, scarcity increases reliance of governments on external sources of funding, even if the funding conditions are misaligned with local priorities.

The PEA included a subset of three counties/districts in each study country where the UNICEF was already supporting health system strengthening efforts. Selection criteria included geography, population demographics, main health priorities, governance structure and health systems performance where data were available (Table 2). County/district selection focused on maximizing variability in order to capture a variety of experiences, thus the final study sites were Garissa, Kisumu and Turkana counties in Kenya; Nkhata Bay, Thyolo and Zomba districts in Malawi; and Kiryandongo, Iganga and Isingiro districts in Uganda.

**Table 2.** Study site characteristics, by country

	**Counties**		
**KENYA**	**Garissa**	**Kisumu**	**Turkana**
Geographical location	Northeast	Western/Lake Region	Northwest
Total population	841 353	1 155 574	926 976
Population characteristics	Nomadic pastoralists	Urban	Nomadic pastoralists
Other characteristics	Conflict—internally displaced persons (IDP), refugees	UHC pilot county	Understudied
	**Districts**		
**MALAWI**	**Nkhata Bay**	**Thyolo**	**Zomba**
Geographical location	North	South	South
Total population (2018)	285 795	721 456	746 724
Other characteristics	Only UNICEF district health strengthening initiative district in Northern Malawi	Less researched; smaller district	Includes urban areas
	**Districts**		
**Uganda**	**Kiryandongo**	**Iganga**	**Isingiro**
Geographical location	West	East	West
Total population (2014)	266 197	504 197	486 360
When the district was established	2010	1975 (name changed in 1980)	
Other characteristics	IDP, refugees	Rural	Rural

### Data sources

Documents reviewed included national legislation related to decentralization, national health policies and strategies, national guidelines for planning, budgeting and financial management and evidence from grey and peer-reviewed literature.

KII respondents included stakeholders from government—both in health and other relevant governance positions—at national and level, development partners, civil society and/or community representatives and the private sector where appropriate (see Table 3). After unexpected, pandemic-related delays, KIIs took place between November 2020 and February 2021 virtually or in-person depending on local coronavirus disease 2019 (COVID-19) restrictions at the time.

**Table 3.** Interview respondents, by country

	Kenya	Malawi	Uganda	Total
Total number of interviews	32	36	44	112
County/District/National				
National level actors	3	8	3	14
Study site 1	10 (Garissa)	10 (Nkhata Bay)	14 (Iganga)	
Study site 2	11 (Kisumu)	9 (Thyolo)	13 (Isingiro)	
Study site 3	8 (Turkana)	9 (Zomba)	14 (Kiryandongo)	
Affiliation				
Government	29	26	39	94
Implementing partner	3	4	5	12
Others	0	6	0	6
Gender				
Female	6	4	7	17
Male	26	32	37	95

Key informants were asked questions regarding health planning, budgeting and implementation, including:

- Local processes, including how and why they differ from established guidelines
- Identification of local health priorities and trade-offs that occur during health planning
- Health systems arrangements, including decentralization and governance, and their impact on health plans
- Actors involved, the relationships and power dynamics between them
- Processes for community engagement as set out in guidelines and in practice
- Suggestions for improvement of the planning process.

We used a combination of standard open-ended questions as well as vignettes to explore these issues (see Supplementary files for sample interview guide).

### Data analysis

#### Qualitative data

**Figure 2.**
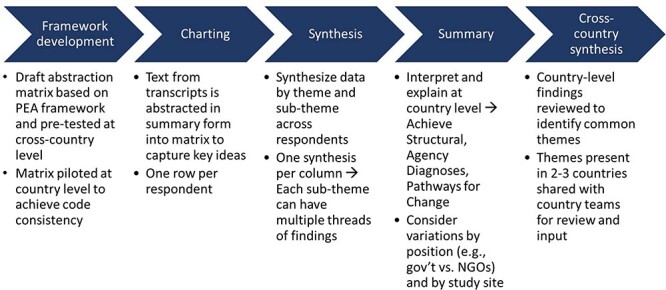
Data analysis steps

Study teams used the Framework Method—a form of thematic analysis—to deductively analyse the qualitative data (Gale *et al.*, 2013), whereby data from KII transcripts were abstracted into a spreadsheet capturing key PEA domains that contained all themes and sub-themes from the theoretical framework (Figure 2). The analytical framework has a matrix of three domains: structural diagnosis, agency diagnosis and pathways for change. Under each domain, there are two to three themes, and each theme has several sub-themes (see Supplementary Materials for the final abstraction matrix and further details). The analytical framework and abstraction matrix were revised through two rounds of testing with transcripts from all three countries, first by the Johns Hopkins University (JHU) team and then jointly between the JHU and country teams, prioritizing consistency and reliability among abstractors.

After the matrix was finalized, each country team proceeded with the data abstraction, known as charting. As each transcript was read, the analyst would generate short summaries of portions of text, which were then abstracted into the relevant cell of the matrix. Each respondent was captured in one spreadsheet row.

Synthesis of data was done thematically across respondents and across counties/districts in each study country. Initial recommendations for overcoming environmental and governance barriers to effective health management were developed through data analysis and then revised and elaborated via stakeholder consultations.

To identify cross-country findings, the JHU team reviewed and captured common themes emerging from country-level syntheses by reviewing individual country reports and study data to identify critical issues and then discussed together iteratively. As common challenges and recommendations were identified, these were presented to country teams for input and review.

#### Budgetary and expenditure analysis

Since the completeness of the budgetary and expenditure data varied by country, each team conducted a core trend analysis of government budgets for each district. Where additional financial information was available, country teams also conducted a trend analysis of expenditures and donor funding trends, which were compared with government funding. Financial data across sites and countries were inconsistent and at times incomplete, so analyses are not presented here (financial analysis is available in individual country reports).

### Rigour

We pursued several approaches to strengthen our methodological rigour. In addition to triangulation between data sources, we conducted regular debriefing within- and between-research teams to review interpretation of results. Second, we conducted member checking by presenting draft results locally in each country to government MoH officials, health management teams, UNICEF staff and others to generate feedback and aid interpretation (see Supplementary Material for more details). Draft recommendations were generated by country teams and then discussed and refined as part of the stakeholder engagement process.

The JHU team synthesized country-specific findings to identify common experiences across countries. These were reviewed and revised in partnership with country study teams. For cross-country results, in particular, we practiced regular reflexivity during analysis.

This study was reviewed by the Institutional Review Board at the JHU Bloomberg School of Public Health. The study was also reviewed and approved by the Amref Ethics and Scientific Review Committee in Kenya, the Higher Degrees, Research and Ethics Committee at Makerere University in Uganda and the University of Malawi Research Ethics Committee in Malawi.

## Results

Given the problem-focused nature of this PEA, we focus on describing the common factors challenging health governance and management across all three countries. The issues described further reflect intersecting challenges related to financing, relationships between actors, bureaucracies and power dynamics; some are connected directly to the health planning and budgeting process, while later challenges reflect broader, more complex issues that go beyond the health sector. Some issues, such as those around funding, likely reflect broader financial management challenges that affect the whole public sector. Although presented in aggregate form, these issues can play out differently in each country or district based on the local context and constraints, which is detailed in more depth in country publications (Munthali *et al.*, 2021; Ssengooba *et al.*, 2021; Tsofa *et al.*, 2023).

We present identified challenges under the domains from the PEA framework: ‘structural diagnosis’ and ‘agency diagnosis’. However, it is important to note that many interact and cut across structural and agency domains, so simple categorizations may be misleading. As our analytical method was problem focused, promising practices and adaptive behaviours are not discussed here.

### Structural diagnosis

Structural diagnosis refers to the structural features or contexts that influence how a problem is shaped, as well as the formal and informal norms, or rules of the game, that shape power dynamics and eventual outcomes. For example, we examined how health planning guidelines were operationalized and interpreted to capture how institutional structures and norms lead to deviations or adaptive processes. The PEA identified three issues here.

First, ‘thick bureaucracy is an unintended consequence of decentralization and constrains decision space’. Decentralization of service delivery and stakeholder engagement has increased the bureaucratic layers at level because the centre has not fully relinquished control, which paralyzes decision-making. The complexity of the health planning and budgeting requirements coupled with short yearly planning cycles leads to impractical health plans and creative coping strategies so that me of which undermine community engagement principles. For example, there is over-reliance on data during health planning in Uganda rather than pursuing community consultations. Rigid procedures hinder adaptive thinking at the level.

Governments generally are a strait jacket. If you try to be creative, thinking that you are doing a noble cause, you will burn your fingers. (UGANDA, Government, PEA_11)

The DHMT is afraid of the political pressure which is coming in and the politicians imposing on the programme. And at the same time he also has to be responsible to the council and citizens who are also demanding something different from what the central level is demanding. (MALAWI, Government, PEA_02)

There is usually a lag period when the IFMIS [Integrated Financial Management System] is closed that slows down your activities – IFMIS is closed for about three months between July and September…you cannot do much until IFMIS is opened and until the [REDACTED for anonymity] county receives funds…July and September is actually at the beginning of financial year so during these three months most of the implementations that we’ll do will be from grants and other implementing partners because remember their money is held separately by them and they manage their own funds so you are able to move those activities. But in terms of government funding implementation of your work plan…always starts somewhere in October, November when IFMIS has been opened. (KENYA, County Manager 1)

Bureaucratic processes which are meant to improve engagement and accountability imposed high transaction costs and counterproductive processes by slowing down the planning, creating unmet expectations and increasing costs. Additional bureaucratic layers have also resulted in more opportunities for weak components of the system to delay or break down the system (i.e. more accountability plus more bottlenecks). In Kenya, bureaucracy is manifest in the large number of committees with overlapping functions, high turnover requiring frequent efforts to orient new participants and limited administrative capacity to identify bottlenecks and improve efficiency. These issues highlight an important distinction between governance and management at level. Planning processes, as demanded by governance structures and regulations, are difficult to manage, navigate and balance successfully, including engagement of different stakeholders.

Second, ‘health planning and budgeting are misaligned because budgets are path dependent and chronically underfunded’. Subnational budget ceilings are typically based on the historical budget of years before with a limited proportional increase (e.g. 3% year on year) rather than budgets that respond to local health plans or shifting health needs. Meanwhile, local health planning follows national guidelines and procedures that must balance community priorities, local government needs and central government mandates. Since planning and budgeting processes and timelines are not well linked, plans often scope activities that are well beyond available budgets.

Also, major health system investments, such as health facility construction and maintenance or increasing the health workforce, are not accounted for under current planning approaches and financial resources because these investments are lacking at all health system levels. The misalignment between planning and budgeting hurts relationships as it leads to frustration and low morale among staff who engage in these cycles every year and creates disappointment and mistrust between staff and communities as community priorities remain unaddressed.

The DHO [district health office] is funded based on the old district hospital structure. As such, the funding doesn’t match with the new structure which has more bed capacity and staff unlike the old hospital. (MALAWI, Government, MW_04)

We may need equipment or renovation, but these remain unfunded because there is always no money and [development] partners do not want to fund such things…they reached an understanding amongst themselves that they do not want to go through capital development. (UGANDA, Government, PEA_07)

In terms of confidence in the [planning] exercise we are slowly losing it… If the ceiling would be brought to us earlier we would then plan as per the ceiling not as per the wish list so that then the people that we are supervising would not get demoralized and demotivated…[by] doing the same thing over and over and they are not getting a change. (KENYA, County Manager 6)

Third, ‘health plan implementation is affected by resource constraints, which results in trade-offs and unfulfilled plans and priorities that deeply undermine health plan implementation’. Examples of constraints include (1) delays in releasing information about budget ceilings during health planning, (2) delayed funding disbursement during implementation, (3) inability to raise funds locally and (4) lack of emergency funds. In Kenya, despite regulations requiring timely disbursements, delays from the (centralized) Exchequer are a well-known and widely reported problem. Underfunding and delayed disbursements in Malawi lead to planned activities not being fully implemented with shifts taking place between flexible activities and inflexible budget lines like utility bills. Plans are then repeated year after year because of incomplete implementation.

…when we do the budget like we are supposed to, [you] get this funding on quarterly basis, but at times you can even get only once in a year. So you wait, wait, wait, wait… (KENYA, County Manager 4)

Actually, there is no financial year that moves in an appropriate and timely manner…that has not happened; all the phases are delayed. (UGANDA, Government, PEA_12)

If you look at AIP - the Annual Implementation Plan 2020-2021, [you’ll notice that] not all issues are accommodated because of resource constraints. (MALAWI, Government, MW_09)

Several key issues exacerbate this problem. First, there is weak accountability for central-level delays in disbursement of funds, but local teams remain bound to the same performance expectations despite delays lasting 2–3 months. Second, most health budgets are restricted or have conditional funds whereby pre-ordained priorities from central MoH result in *de facto* decision-making on spending and limit local-level discretion for implementation. Third, administrative capacity and discretion to manage these constraints are lacking. In Uganda, DHMTs face strict sanctions such as loss of monies if districts fail to use and account for the disbursed funds by set deadlines every quarter. Delays to disburse and pressure to account for funds create dysfunctional coping behaviours such as super-short implementation timeframes. Many respondents also complained about conditional priorities and unrealistic performance expectations handed down from the centre to the local level, as well as mismatched targets compared with resources made available.

### Agency diagnosis

The agency diagnosis refers to the relationship and power dynamics of actors, and the motivations and interests that incentivize their behaviour. For example, we explored how health management teams juggle their relationships with government officials and development partners at local level as well as central ministry officials. Here, we discuss four emergent themes.

First, ‘shifting power dynamics create ambiguity and constrain local decision-making’. Recent changes linked to decentralization create uncertainty, ambiguity and distress among actors whose roles and responsibilities are changing individually and in relation to other actors in the system. These shifts affect how different actors see and enforce their role in health planning. For example, in Kenya, there are many new mandates and actors involved, but clarity on roles and responsibilities for planning and budgeting is lacking, partly due to high turnover and increased presence of political actors and citizens under the post-2013 devolved government structure.

CAO [Chief Administrative Officer] is the most influential actor at the district level. He controls all the money. (UGANDA, Government, PEA_ 08)

If you look at this place, it is big but we have to ask everything from the district and then they have to decide okay this we can buy, this we cannot. So yes, they may go by our priorities but not always. We are not able to immediately respond to certain important areas because we are not the ones to procure or to make decisions about procurement. (MALAWI, Health facility manager, MW_02)

The system gives a lot of power to the political leadership at that level, but these political leaders do not have enough understanding of the planning or budgeting process. (UGANDA, Government, PEA_11)

Importantly, the cycle of demands and accountability between health managers, government and central MoHs are not bi/multi-directional. This means not all actors face the same pressures, and health managers often feel powerless vis a vis ‘both’ local and central government actors with limited meaningful support. In Uganda and Malawi, e.g. DHMTs are facing substantially more pressure from many actors over which they have little equivalent power. This leaves DHMTs feeling obligated to follow mandates from central MoH even if they are not aligned with local priorities and disempowered to negotiate between district government and MoH.

The DC [District Commissioner] says ‘I am the president of this district, I don’t want to hear that somebody has done such and such things without my knowledge’ So, we can say we don’t have full freedom. (MALAWI, Government, MW_05)

The DHMT is afraid of the political pressure which is coming in and the politicians imposing on the programme. And at the same time, he also has to be responsible to the [District] council and citizens who are also demanding something different from what the central level [MoH] is demanding…They don’t know which way to go because they would actually be asked why they did this when this was not part of the priority plans. (MALAWI, Government, MW_02)

Second, ‘meaningful community engagement is uneven’. Community engagement guidelines exist within planning guidelines, but their interpretation and adherence vary widely. Current demands for community engagement require health management teams to engage with many different actors, which is impractical and inefficient to carry out fully. Community engagement in Kenya varies by county due to issues like absent or inactive facility committees (sometimes related to delayed funding) or poor attendance from community members because engagement efforts were treated as a perfunctory task by officials. In Uganda, respondents considered guidelines for community engagement vague and intensive, leaving district officials to develop shortcuts to claim community engagement in planning even when community members are not earnestly or consistently engaged, leading to mistrust between communities and government. In Malawi, district officials face an exceedingly long list of community actors they are supposed to engage (i.e. more than 70 entities); in addition, some expressed negative attitudes about community members’ ability to understand and engage with the planning process, which may influence how they approach engagement.

Sometimes we sit and think that these health management committees are very powerful committees that make things work but in reality not much… the DHO [district health officer], yes. He can crack the whip and do anything but as the health management committee, they cannot go beyond recommending, and even what they recommend is crafted probably by the medical officer or the in-charge or whoever and it may not be really biting. (UGANDA, Government, PEA_11)

I think the technical people should think of building the capacity for people to understand how they can facilitate the process of change at community level. …Then we will see that their village action plans are very inclusive, they will have cross cutting issues in there. Let them understand that, if they don’t understand they will not even reflect that in there planning. (MALAWI, NGO, PEA-10)

Other challenges are balancing community priorities with competing factors, such as mandates from central MoH in Uganda or limited budgets in Malawi, whereas in Kenya, community engagement mandates come with dedicated lines of financial support. From a transparency standpoint, final health plans and budgets are not consistently publicized or available in places and language where community actors can understand them. Health facility providers, as a key constituency, could be more meaningfully involved in health planning. All these factors jeopardize relationships with community actors in the long run and risk leaving the community priorities insufficiently addressed.

They take our problems and present them to the ADC [Area Development Committee]. And the ADC compiles and forward them to the district council. But we do not get feedback on most of the problems presented to them…As such, most people are reluctant to present their problems/needs to the ADC knowing that nothing will happen. This is a very big problem. We don’t know whether because there is no money, but it’s even hard to know if the money meant for a particular project is available. (MALAWI, Civil society, MW_09)

Third, ‘harmonization and accountability mechanisms for donors, their implementing partners, and country actors (at national and levels) are dysfunctional, which affects planning and budgeting’. Despite the existence of development partner harmonization mechanisms at level, such as health stakeholders’ forums, or memorandum of understanding requirements, these are not functional nor reliably used or enforced. Development partner investments are still typically negotiated at the national level, without substantive involvement of governments. This type of arrangements leaves gaps in accountability for development partner investments and can result in misalignment with local priorities or in non-compliance with local requirements. Local governments face difficulties enforcing harmonization of development partner programming to local priorities for fear of jeopardizing external funding, especially when programming negotiations have taken place at the national level between central government and implementers.

There is also tension around development partner–funded activities which tend to be managed vertically. On the one hand, development partners provide stopgap funding for unmet needs/unfunded priorities at level; however, the lack of coordination between development partners and the lack of transparency around their budgets and timelines mean that their support is seen as unreliable, less aligned to plans and unlikely to sustain programmes. These dynamics create difficulties in developing and implementing health plans that are comprehensive. It is important to note that these issues are partially promoted by inconsistent harmonization and accountability expectations at different levels of the health system, such as when national mechanisms are not strictly enforced, or central agreements are made in opposition to local priorities.

But our funding does not allow that [direct cash transfer to districts]. While we do budget support, it is in kind. We will not put money on the district account…if it [is] the health worker who have done the activity, we send the money directly to that health worker. (UGANDA, Implementing Partner, PEA_19)

Most of them [implementing partners] do not disclose. They will say we are going to do this, but you may not know about the budget, how much they must spend. (UGANDA, Government, PEA_33)

The other issue is on budgets from partners. They come here, we don’t know their budget and they just start implementing their activities, which is not supposed to be like that. We have to know that this partner is coming here, and they have this amount of money and these are their activities. (MALAWI, Government, MW_05)

Finally, ‘insufficient public administration capacity exacerbates other challenges in health planning and budgeting’. Administrative and managerial capacity for public administration at level is lacking both in number and expertise across the public sector. As decentralization and bureaucracy advance, the burden on administrators grows beyond the capacities of available staff, which is exacerbated by turnover. Respondents highlighted how awareness of health sector needs among local government staff outside of health could contribute to better planning and implementation as others within the local ecosystem understand health system needs better.

[There are] two challenges; one is the limited guidelines or limited supportive supervision from the national level for the counties to do proper evidence-based planning, and then second challenge is the capacity at the county level to do this type of analysis. (KENYA, National Government 3)

….at [District] council level we face some problems because the head of finance sometimes may not understand problems at the district hospital in terms of health management. The way we prioritize our activities here, somebody who controls funds at the Secretariat may not be conversant with our priorities. (MALAWI, Government, MW_01)

Another capacity example is around barriers to accessing data systems either because of limited technical capacity of staff or to limit corruption (e.g. in-person manual entry for Kenya’s budget management system), which leads to choke points. For example, procurement at district level in Malawi relies on a few procurement officers who are responsible for serving all the district’s procurement needs, leading to delays. Furthermore, when only one person has access to health monitoring and financial data systems, it leads to overburdening that person and/or centralizing power in them which can be difficult to counter.

The arrangement of the health system is okay, but it is paralyzed because of financial problems. It delays the whole process because we have one controlling officer. For the things to be processed, once the DC [district commissioner] is out, nothing moves. We wait for the DC, yet the DC is supposed to attend meetings. Once he goes for a meeting for a week, then there is a standstill in the processing of whatever in terms of funding. (MALAWI, Government, MW_03)

It is critically important to note that while capacity needs are connected to and contribute to the other challenges highlighted here, addressing these capacity gaps is not enough to resolve the other problematic dynamics at play.

### COVID-19

Our study’s data collection took place during the first year of the ongoing pandemic, while local health teams were juggling implementation of previously approved health plans along with pandemic response. Although not focused on COVID-19’s impact on health teams, a few themes emerged. Unsurprisingly, responding to COVID-19 placed additional pressures on health management teams. COVID-19 negatively impacted health plan implementation due to more severely constrained budgets and implementing new priority activities linked to surveillance and quarantine. However, the pandemic response, at times, improved relations between teams and central government through increased communications and/or increased funding with more latitude to craft appropriate responses to deliver on COVID-19 mandates.

There have been more interactions now with the central level with the coming of the COVID. They have been visiting and engaging us frequently, holding meetings with them. So you will find that it has improved our interactions. It is very different with what used to happen before that. (MALAWI, Government, MW_02)

Of course, when the [COVID-19] outbreak happened budgets had already been passed…at least for some counties they were able to pass supplementary to allocate to various to enable response. I know even from the national level went to assist the county legislatures who also had to pass supplementary to allow any expenditure to happen…When you think of Kenya of course those supplementary were basically reallocations from the other areas …for instance it mostly affected some of the planned activities for UHC scale up. (KENYA, National Government 2)

### Pathways to change

Problem-driven PEA focuses on the political economy factors that influence the problem at hand, thus setting the basis for an interventionist approach to addressing any challenges identified. As mentioned earlier, study teams presented and refined potential recommendations with local stakeholders, including considerations about timing. Table 4 presents five recommendations to better support health governance and management for the short, medium and long term that cut across all three countries. These recommendations address issues around managerial capacity, engagement and accountability between health teams and communities, coordination and accountability between government and development partners, innovations in financing at level and addressing the need for large-scale health system investments. Each recommendation includes a brief description, examples of potential actions drawn from country studies, additional changes to social norms, policies or resources that would facilitate success and key considerations and threats.

**Table 4.** Recommendations for supporting health governance and management

Recommendation	Details + Examples	Timeline	Changes needed	Considerations and threats
Build capacity for planning, budgeting and management at level	Capacity building at level is needed not just for the mechanics of health planning, budgeting and implementation but also around actor management and negotiation. Topics should intentionally include soft skills and political analysis skills to analyse local contexts and how to leverage these for improved resource allocation for the health sector. Efforts to improve confidence of managers to stand up to pressures would also be helpful. Capacity building efforts should address connections between health sector planning and budgeting and the rest of government, especially other large sectors, and target both health and broader government actors. Taking decentralization dynamics into consideration is critical, especially in Malawi and Uganda. Given their role managing implementation of service delivery targets, consider including health facility managers and service providers in capacity building efforts	Short term	Social norms around allowing more localized decision-making and discretion at level	Addressing capacity needs is insufficient for addressing broader relationship and environmental factors that constrain management teams
Revisit engagement and accountability mechanisms between government and communities to make them more practicable and productive	Current community engagement mechanisms are not practical given constraints on health management teams, leading to mistrust and disconnection between communities and health officials. Strengthening community engagement should focus on minimizing the potential for broken promises. Community engagement guidelines could be revisited to pursue simplification and/or prioritization of consultations to ensure more meaningful engagement. Actor roles could also be reviewed to determine where engagement should be pursued by managers (e.g. DHMTs) and where they could be delegated to others, like facility committees. EXAMPLES Some settings need better support for continued community engagement in planning, incl. dedicated funding. Other settings need reducing the engagement requirements necessary for each yearly planning cycle. Greater transparency about planning process, incl. constraints associated with limited budgets. Improve dissemination of health plans and budgets to facilities and communities. Greater engagement of service providers and health facilities both within the planning process and as a conduit for community priorities.	Short-to-medium term	Policies to allocate funding to support community engagement and make community engagement requirements more flexible. Social norms around: Prioritizing community engagement Relationship with government	Local and national government support are necessary to have long-term effects
Strengthen coordination and accountability between government actors and development partners	Coordination and accountability between government actors and development partners, including donors and implementers, are complex and existing mechanisms need to be revisited and strengthened. It is also critical to address the relationship and power dynamics within government around national governments negotiating with development partners on programs to be implemented at level with little input from government actors. EXAMPLES Strengthen and support implementation of the ‘Sector Wide Approach’ principles of joint planning, joint implementation and joint monitoring framework between government and partners. Support implementation of existing donor coordination mechanisms at local and central level, including strengthening accountability mechanisms and creating need-based and equity-oriented distribution of donor-funded programs Establish procedures and consequences for development partners who plan or implement projects that are not aligned with local priorities (e.g. government oversight/approval of NGO activities, refusal of future projects for non-compliance). Strengthen dialogue between national and government actors regarding coordination of development partner investments and alignment with local priorities. Improve transparency of external funding at level. Channel donor funding for systems development to governments rather than through third-party contractors. Improve development partner engagement in planning and budgeting	Short-to-medium term	Social norms around the transparency and mutual accountability between development partners and local governments. Policies to introduce new accountability mechanisms, modify existing ones and implement effectively	Requires buy-in from government actors to enforce existing accountability mechanisms and/or introduce new ones. National government may be resistant to relinquishing influence over local-level development projects. Development partners will likely be resistant
Support innovations at level to address financial constraints on health planning and budgeting	Beyond potential strategies for revenue generation that would increase local budgets, funding constraints at local level would benefit from creative thinking and piloting of potential innovations to address delays and shortfalls. This is connected to issues of transparency and bidirectional accountability within the public sector and is especially salient in contexts where central budget transfers are delayed, leaving managers to struggle with financing health plan implementation. Further engagement with experts in public financial management would be critical. EXAMPLES Earlier announcement of budget ceiling from central government. Examine legislative limits or administrative controls on borrowing to allow for urgent resource mobilization. Improve resource tracking between sectors at level to improve transparency in allocations. Options include establishing separate accounts or sub-accounts for large sectors (e.g. agriculture, education and health). Establish emergency procurement processes	Medium-to-long term	Policies (laws, regulations) to permit greater flexibility for financial management and resource mobilization Social norms around innovation in local financing and cost sharing	Requires advocacy and involvement from government actors
Develop framework for major health systems investments	Large-scale health systems investments, like infrastructure, maintenance and substantial increases in health workforce, are currently underfunded and competing with recurrent costs. Worse still, current prioritization efforts can be co-opted by politicians who want electoral gains from infrastructure spending. A coordinated and strategic framework that outlines key health systems investments and how they will be prioritized and financed should be negotiated between national and health system actors to reduce the burden on local budgets for major investments. EXAMPLES Advocate for donor investments on infrastructure. Support establishment of crisis funds that do not draw away from yearly health plans. Introduce limits to insulate health systems investments from political influence, such as targets by catchment area or by burden of disease	Medium-to-long term	Policies that direct funding allocations to health system infrastructure. Large-scale financial resources to directly fund health system infrastructure	May face resistance from national government, including elected officials who want to retain influence and development partners who are unable or unwilling to cede control

We recognize that not every aspect of these strategies will appear feasible or politically palatable, but they reflect the priorities that local stakeholders identify as necessary. Several of the challenges identified earlier, such as bureaucracy and power dynamics, are complex—sometimes intractable—systemic issues linked to governance that are beyond the scope of any one actor or project and require leadership and engagement from government actors at multiple levels. The recommendations outlined here are intended to make small improvements in this space to make the overall system more functional. Importantly, these recommendations are presented in generic form and would require tailoring to the national and context to address specific issues to be most effective (see also country-specific publications).

## Discussion

This PEA highlights several critical and complex issues that require attention to further strengthen health management and governance. First, we found that unfinished reforms or partial ownership of new responsibilities under decentralization hinder effective management. Incomplete decentralization creates unhelpful ambiguities around roles and unclear processes, which often result in additional layers of bureaucracy that drain time, resources and energy. This is coupled with prevailing information asymmetry between local, district and national level which creates misalignment in terms of priorities. Under these circumstances, health management systems for planning, budgeting, information use and procurement sometimes do not link or reinforce one another. In terms of community engagement, Wilson describes how complex organizations develop rules to constrain its managers into complying with ‘claims of constituents’, but rules can often multiply to the point of inaction as we found around community engagement guidelines (Wilson, 1989). As decentralization rolls out, management systems should be reviewed to ensure they are fit-for-purpose and aid managers in the achievement of their aims. Across all three countries, efforts to reduce complexity and bureaucratic demands through simplification and alignment of processes and systems could alleviate pressure at the local level.

Second, governments develop detailed health plans that are rarely fully funded. As noted elsewhere, from overall government expenditure on health, to intergovernmental fiscal transfers to the level, to health-specific allocations at the local level, budgets remain too small to meet the needs of mandated responsibilities under decentralization (Masaba *et al.*, 2020; Mansour *et al.*, 2022) or to effectively deliver on PHC local priorities (Hanson *et al.*, 2022). While there is room for aspirational planning, the reality is that underfunding leads to uncomfortable—and at times, inequitable—trade-offs that permeate governance and management. External funding can provide an important stopgap for constrained budgets, but donor funding earmarks are often mismatched with local priorities. Achieving UHC requires that fundholders also radically rethink their operating models. Health systems hardware, such as infrastructure and human resources, also remain an important and underfinanced need. The ongoing COVID-19 pandemic has exposed the vulnerability of local health systems to disruption making it more critical than ever to invest in health systems capacity to deliver PHC services and public health functions and be prepared for resilient crisis response.

Third, the operating environment in which managers work is often counterproductive, thus disincentivizing good management practice. There is a persistent disconnection between the demands of local constituencies, the resources available and managers’ decision-making authority, leaving them in uncomfortable positions that require considerable motivation and skill to navigate. These findings align with Bulthuis *et al.*’s work in Malawi and Uganda which point to the dispositional power exercised by central MoHs by virtue of their role in the health sector hierarchy and their ability to limit or control budget priorities through earmarks, e.g. conditional grants. These dynamics combined with the influence exerted by local political bodies limit decision space for health managers, which is evident in our results (Bulthuis *et al.*, 2021). This tension could be improved by expanding both the PHC resource base and health managers’ decision space, so they can negotiate priorities and trade-offs with local communities and stand by agreements, limiting the risk that national-level decisions may change arrangements. We witnessed improved dynamics during the COVID-19 response where, as coordination between levels of government improved, teams were given more leeway to act and the resource base increased.

We also echo weaknesses in public administration capacity for health planning and budgeting identified earlier. For example, despite having dispositional power over the health sector based on clinical knowledge, DHMTs in Malawi, Uganda and elsewhere have been shown to be constrained by their lack of knowledge and skills around political lobbying and advocacy, policy comprehension and project management (Bulthuis *et al.*, 2021). In Kenya, counties’ eventual ability to effectively assert newfound decision space required time to build capacity and structures for effective management as well as clarification of roles and responsibilities (Tsofa *et al.*, 2017a,b). Findings elsewhere point to the synergistic links between capacity for district-level planning functions and decision space for health managers, whereby managers with greater capacity to take decisions have a foundation to exercise greater scope in their decisions (and vice versa) (Bossert and Mitchell, 2011), which suggests important links between capacity and independent decision-making in our study areas also. National commitment to investment in health leadership and administrative competencies among health managers could further refine the soft skills required to manage this complexity, as has been suggested by others (Gilson, 2016).

Champions of PHC reorientation have acknowledged that it must be a whole-society approach, which is fundamentally political and requires long-term commitment (WHO & UNICEF, 2018b). In other LMICs, inadequate financing and challenges with decentralization have already been identified as challenges to achievements in maternal and child health (Hipgrave *et al.*, 2019) and to PHC (Langlois *et al.*, 2020). We highlight the complex issues raised by this study so that they can be considered as countries negotiating new pathways for achieving PHC aims, including renewed consideration of health management and governance arrangements that create greater opportunities for leadership. Research on public financial management (PFM) in South Africa suggests that authentic collaboration and embeddedness between financial management and service delivery is necessary not only to build trust but also to develop PFM strategies that are realistic (Wishnia and Goudge, 2021). Because these issues are beyond the scope of the health sector alone, and technical, short-term fixes will only be met with limited success, longer-term reform is likely needed as highlighted by the recommended pathways to change related to accountability and financial envelopes.

### Limitations

This study is subject to several limitations. First, each country included three study sites, which limits the generalizability of the findings at the country level. However, stakeholder feedback suggests that these issues are common and prevalent across all districts and/or counties. Furthermore, this paper focuses on common themes across countries, so contextual differences are missing but can be found elsewhere (Munthali *et al.*, 2021; Ssengooba *et al.*, 2021; Tsofa *et al.*, 2023). Second, despite overlapping with the emergence of COVID-19, our study did not intentionally set out to assess the impact of the pandemic on health planning, budgeting and implementation. Any findings in this aspect were incidental, emergent and warrant careful interpretation. Third, and relatedly, the persistence of COVID-19 has potentially changed some of the historic dynamics described here. Certainly, the improved flexibility and communication resulting from the pandemic response are adaptations we hope carried forward into the future. Finally, we identified two key challenges with PEA methodology. PEA requires a standard problem statement and outcomes, so for a cross-site, cross-country study, this can lead to an overly broad problem statement. Furthermore, we found some of the concepts in the PEA framework difficult to apply at times. For example, ‘credible commitment’ or ‘mental bias’ was difficult to define or identify in the data requiring inferences by analysts that went beyond respondents’ actual words.

## Conclusion

An Achilles heel of the first global push for PHC in the 1970s and 80s was insufficient health management and governance capacity to support health system reorientation. In principle, national public sector organizations recognize the value of decentralization; in practice, they retain decision-making authority centrally for fear that local managers’ decisions will reflect poorly on central authorities (Wilson, 1989). This study illustrates the importance of taking into account the systems, processes and institutional arrangements that affect management and governance beyond the investments needed to support PHC. It also highlights the conflicting role of development partners engaging at the level, especially when there is limited accountability to and alignment with local priorities. National policymakers’ and development partners’ engagement with the political dynamics and enabling environment, including those beyond the health sector, will be necessary to support health management and meaningfully expand PHC in pursuit of the SDGs by 2030.

## Supplementary Material

czad021_Supp

## Data Availability

The data underlying this article cannot be shared publicly to protect the privacy of individuals who participated in the study. Data can be made available upon reasonable request to the corresponding author.

## Reflexivity statement

This article presents research conducted by a diverse group of researchers in terms of gender identity, seniority and citizenship. This article includes nine authors who identify as women and 11 who identify as men. Eight of the authors are early career researchers. Six of the authors are affiliated with high-income country institutions, while the remainder are affiliated with LMIC institutions. The majority of the research and writing team includes persons who are citizens and/or residents or have significant lived experiences in LMICs, including the three countries included in this research. Several team members also have experience working for MoHs, including at level, which brings additional perspectives to the research. The research team has multidisciplinary expertise in economics, policy analysis, clinical medicine, public health and social science. All authors have extensive experience with qualitative research and health policy and systems dynamics in the Global South.
