# Supplementary material for: Political economy analysis of subnational health management in Kenya, Malawi and Uganda
Source: Health Policy Plan. 2023 Apr 11;38(5):631–47. doi: 10.1093/heapol/czad021 (PMC10190959; doi:10.1093/heapol/czad021)
Supplement: czad021_Supp [file czad021_supp.zip › UNICEF PEA Cross-Country Paper_SUPPL MATERIAL_2022 08 18.docx]

# Political economy analysis of sub-national health management in Kenya, Malawi, and Uganda

SUPPLEMENTARY MATERIAL

In the first round of testing, the [REDACTED] team tested the abstraction matrix using transcripts from all three countries. The team reviewed the matrices for consistency, discussed reliability among abstractors, and modified the matrix and its related themes and sub-themes accordingly. Based on this initial round, the [REDACTED] team developed a pre-recorded training session for country teams – orienting them to the framework and the overall data analysis process. The training was shared online for asynchronous viewing then reviewed during a cross-country call, allowing for discussion and clarifications. In the second round of testing, each country analysis team tested the abstraction matrix with a transcript, discussed emerging questions, and made a final round of minor adjustments to the matrix.

In the matrix, teams added one synthesis summary per column, so that each sub-theme captured multiple threads of findings. Country teams held internal meetings to discuss emerging themes and to interpret and explain the data.

Abstraction Matrix

| STRUCTURAL DIAGNOSIS | | AGENCY DIAGNOSIS | | | CHANGE | |
| --- | --- | --- | --- | --- | --- | --- |
| Structural Features *(Contextual factors that impact the problem)* | Rules of the game *(Institutions and rules that shape power dynamics and outcomes)* | Actors *(Relationships and balance of power between them)* | Motivations *(Factors that shape actor/org behavior)* | Concepts *(Factors that clarify actors' incentives and decisions)* | Implementation *(Factors relating to the implementation of health sector plans)* | Pathways for change *(Plausible pathways and actions for intervening)* |
| Politico-econ structures (e.g., elections, economic conditions) | Application of formal guidance in practice | Actors playing decision-making role at local level | Motivations that affect the problem | Credible commitments vs. empty promises | Implementation process | Leverage points to precipitate reform |
| Historical legacies re: governance and community responsiveness | Formal vs. informal rules (e.g., regulations vs. social or org norms) | Power of/between actors at local level | Motivations that affect potential reform | Path dependence | Extent to which health plan is implemented | Strategies or activities to bring about reform |
| Socio-cultural structures (e.g., trust in gov't, gender, and other demographics) | Stakeholder engagement process | Relationships between actors, incl. between nat'l and local | Equity orientation | Information asymmetry | Examples of programmatic success | Champions or Critics of potential reform |
| Health system organization and funding (e.g., decentralization) | Decision-making process |  | HS responsiveness to local needs | Biases and mental shortcuts | Examples of programmatic challenges/conflicts | Timeline for reform |
| COVID-19 | Transparency in health planning |  | Health priorities | Corruption and rent-seeking |  |  |
|  | Accountability mechanisms, incl. supportive and punitive |  | Trade-offs between priorities and how they are made | Rank/hierarchy |  |  |
|  | Discretion |  |  |  |  |  |

PEA Dissemination Activities, by study country

|  | *Kenya* | *Malawi* | *Uganda* |
| --- | --- | --- | --- |
| UNICEF country office | Virtual dissemination with UNICEF-Kenya Zonal officers. | Virtual dissemination with UNICEF-Malawi and CHAI. | Virtual dissemination with UNICEF-Uganda. |
| Study sites | Shared summary briefs with county directors of study counties. | In-person meetings with district health management teams and others in each study district. | District-level meetings with district officials, health workers, implementing partners and others in two out of three study districts. |
| National level | Virtual meeting with Department of Health and Ministry of Health stakeholders. | Central-level validation meeting with Ministry of Health, Ministry of Local Government and Rural Development and others.  Shared country report with other government stakeholders and implementing partners. | Present findings at national-level budget meeting, with UNICEF representation.  Shared country report with other government stakeholders and implementing partners. |
