# Supplementary material for: Political economy analysis of subnational health management in Kenya, Malawi and Uganda
Source: Health Policy Plan. 2023 Apr 11;38(5):631–47. doi: 10.1093/heapol/czad021 (PMC10190959; doi:10.1093/heapol/czad021)
Supplement: czad021_Supp [file czad021_supp.zip › UNICEF PEA IDI Guide_Aug 2020 GENERIC.docx]

Political Economy Analysis of sub-national health management in Eastern and Southern Africa

In-depth Interview Guide

V3 – 5 August 2020

# INTRODUCTION

Thank you for agreeing to meet us.

We are conducting a political economy analysis of health management and governance in [KENYA/MALAWI/UGANDA]. Our goal is to understand how the policy environment, health systems context, relationships between actors, and structures for accountability influence how decisions about health are made at the [DISTRICT/COUNTY] level. We also seek to understand the dynamics between central and sub-national levels. We aim to identify potential areas for change to strengthen health management at the local level.

As part of our work, we are conducting interviews with individuals, like yourself, who are familiar with health management and governance at the sub-national level in [KENYA/MALAWI/UGANDA]. During this interview we will ask you questions and present scenarios about how health issues are prioritized at the [DISTRICT/COUNTY] level, how [DISTRICT/COUNTY] health planning and implementation processes play out in practice, how accountability structures for health work, how the context affects local level health planning, and what potential pathways for change already exist.

| Name of Organization |  |
| --- | --- |
| Your name |  |
| Designation |  |
| Work Area |  |
| Postal address |  |
| Telephone |  |
| E-mail address |  |

**OBTAIN INFORMED CONSENT**

*NOTE TO INTERVIEWER: This is a guide to the interview. You should cover* ***all the main numbered questions*** *in this interview form. You should use the probes selectively, according to the type of knowledge that the respondent conveys, and what you have already found out from documents and other interviews.*

# INTERVIEW GUIDE START

1. **First, can you please describe your role at [RESPONDENT ORGANIZATION]?**
2. **What are the top 5 health priorities in this district?**
   1. Has COVID-19 changed these priorities? If so, why?
   2. What are the outstanding programs in the district?
      1. Were these different before COVID-19? If so, why?
   3. Can you give me an example of an accomplishment you are proud of?
      1. How were you able to achieve this?

# HEALTH PLANNING PROCESS

*INTERVIEWER GUIDANCE: Anything already captured in the document review can be skipped here. Talk respondents through the figure of district planning process as you go. Only use follow-up questions or probes if respondent does not address topic directly.*

**This picture shows how health planning processes at the district level are meant to work. Thinking about what happens in this district/county:**

1. **Which parts of this picture represent accurately what happens in this district?**
   1. How do processes in this district differ from this picture?
   2. Why do you think the processes in this district differ from the picture?

PROBE: timelines and feasibility; powerful actors cutting out others, etc.

- 1. What are the main equity considerations during health planning for the district? E.g. needs of the most vulnerable, burden of disease, available budget, etc.
  2. When you are developing annual health plans and budgets, what kind of information do you draw upon?
     1. How would you describe the information that you use/is available to you? E.g. data are of poor quality, not regularly updated, important information missing, etc.
     2. How helpful is the guidance you receive from the central MOH for using data for planning?
  3. How is information about the annual plan and budget allocations made available to the public?

PROBE: platforms / channels of communication and dissemination

- 1. What are the most challenging parts of developing the annual health plan and budget? Why?
     1. How do these challenges vary year-to-year?
     2. Why do some persist?
     3. Has COVID-19 changed things? Why/why not?
  2. How is COVID-19 changing things this planning cycle?

1. **Thinking about your typical planning and budgeting process, which items keep recurring in each district plan in every planning cycle? Why?**
   1. Which are the new items that emerged as priorities recently? Why?
   2. Which activities or budget items are consistently neglected or under-funded during planning? Why?
      1. What could be done to make them funded priorities?
   3. How transparent is the budget allocation process? Why?
      1. How is this process monitored and by whom?
      2. How predictable are the budgets promised by the central level?
2. **How does the overall arrangement of the health system affect how you make and implement annual health plans?**
3. How does decentralization/devolution affect how you do things? e.g. more/less flexibility, more/less support, etc.
4. Are there issues with the overall health system that limit what you can do? e.g. HR appointments and transfer, on-time funding flows from central level, etc.
5. How does the central-level MOH support or limit what you can do?
6. What does the central level think the challenges are in your district?
7. What do you think are the challenges that the central level is facing?
8. Do you think the issues you are describing about the health system’s organization are experienced in a similar way in other districts? Why/why not?
9. **How would you describe the relationship between the district and central levels of the health system?**
10. Does it vary by office at the central level? e.g. budget office vs. program-specific like MCH.
11. Are there aspects about this relationship that make it difficult to manage the district’s health system?
12. How has the COVID-19 crisis changed the relationship between the district and central levels? Any examples?
13. **To what extent are community members involved in developing health plans and budgets? e.g. assessments, planning, budgeting, implementation, monitoring.**
14. What are the processes in place to facilitate community participation?
15. Are these formal, informal?
16. Any existing structures and guidance?
17. What challenges have been encountered in these processes?
18. What are some mechanisms that the district could use to understand and incorporate community priorities in their health planning?
19. How responsive is the health system to local community needs and demands?
20. **How does the work of non-governmental stakeholders influence the choices you make for the annual health plans?**

PROBE: NGOs, implementing partners or development partners, private sector actors.

1. What challenges do you face in engaging government and non-government actors into the planning process?

PROBE: issues of transparency about budgets, negotiation for priorities and information sharing, dominance of discussions and priority choice, etc.

1. How can these challenges be mitigated?

# STAKEHOLDERS

*INTERVIEWER GUIDANCE: Spread out and go through the list of cards (e.g.* *District health team, facility in-charges, district government, sub-district managers, development partners, national agencies like MOH, community members, NGO or implementing partners.). Make a note of any additions and removals by the respondent.*

**Now I would like to talk about stakeholders. Here is a set of actors who might influence health sector programs and priorities in this district.**

1. **Do all of these stakeholders influence health sector programs and priorities in this district?**

*INTERVIEWER: Remove any cards that the respondent does not think is relevant.*

- 1. Are there any stakeholders missing from this set?

*INTERVIEWER: Add in any missing names onto additional cards*

- 1. Who are the key players in the health planning process?
     1. Who leads the health planning process?
     2. What is role of other actors [for those not leading]?
  2. Have these actors always been involved? How has this changed over time?
  3. Do all of the different actors involved in health planning have the same priorities when deciding what is done in the district for health?
     1. If not, how do they differ? Why?
     2. How do different priorities get balanced?

1. **Now let’s talk about a specific issue – for example if the district were building a new primary health center and was trying to determine where to locate it [OR INSERT OTHER EXAMPLE AS RELEVANT], who would be the most influential stakeholder for that? Who would be the least?**
   1. Why do you think this is?
   2. In general, do you think those same stakeholders would be the most influential in other situations too? Why?
   3. Can you think of a specific example when different actors disagreed over priorities in the annual health plan?
2. How was the disagreement resolved? If the disagreement was not resolved, why not?

# HEALTH PLAN IMPLEMENTATION

**We’ve been talking about how health plans are made but now I’d like to talk about how they are implemented.**

**VIGNETTE #1**

I am going to read a scenario about an imaginary district. I would like to ask you a few questions about how you think people would respond to this.

Imagine that Xanadu is a district in your country. This year their health budget was approved for 15% [OR CURRENCY] less than the DHMT had requested. The program with the largest cut was immunization [OR INSERT OTHER PROGRAM AREA]. The DHMT still has to meet immunization targets for all children in the district.

What would a typical DHMT do in this situation?

What would the district council/gov’t to do in this situation?

What would community members expect the district leadership to do in this situation?

The DHMT has reached out to the head of the Immunization program in [CAPITAL CITY] to ask for more money. They also reached out to some NGOs and development partners that have child health programs in their district for help.

Would any of these stakeholders come through with support to help the DHMT deliver the immunization program as planned? Why/why not?

Two months later, the DHMT found out that the NGOs were actually implementing immunization activities in the district. The DHMT was not aware that these NGO programs were in place. When they talked to the NGOs to learn more about the activities, they realized that the NGO activities were targeting some of the same areas where the district was working, which meant they were duplicating effort.

- What would the DHMT do now?
- Have there been any examples like this in your district that you could tell me about? How did that work out?
- How could a situation like this be improved or avoided? Would these be easy or hard things to do?

**VIGNETTE #2:**

Imagine that Mary is a nurse and a member of the DHMT in Xanadu, a district in your country. The budget and plan that was submitted for the district included funding to support her team’s supervisory visits to community health workers (CHWs) across the district. She thought that this budget and plan were approved, but when she goes to the District Health Officer at the beginning of the year and asks his permission to start supervisory visits, he says that he has no money to support this as the District Council has taken a cut off of his budget. Mary is upset because this is the second year in a row that funding for supervision has been cut from the budget.

- What do you think Mary would do about this?

Later, Mary is talking with a community member from the health facility management committee [INSERT NAME FOR RELEVANT COMMITTEE], and they learn that the District council has taken the money for CHW supervision again. The community member is surprised about this because the committee had received reassurances that the supervision budget would be funded this year.

- How do you think they would react?
- Would they do anything in response to this information?
- Have there been any examples like this in your district that you could tell me about? How did that work out?
- How could a situation like this be improved or avoided? Would these be easy or hard things to do?

# MONITORING and ACCOUNTABILITY

**VIGNETTE #3:**

Now I am going to read out a scenario about decision-making in Xanadu, the imaginary district, then I will ask you a few questions about how you think people would respond to this.

Two years ago, the Xanadu DHMT were told by the central MOH that they needed to include activities for a new program for [INSERT TOPIC HERE]. Although this topic is a big priority at the national level, it is not as important in Xanadu where other health issues present a greater burden on the population. The DHMT knows that they need to include something in their health plan for this new program or they will get a call from the Deputy Health Minister otherwise. But the District Council [INSERT LOCAL GOV’T NAME] will also ask questions when they review the plan and budget.

- What would the DHMT do in this situation?
- Why is the DHMT struggling with this issue?

The district health plan includes activities for the new [TOPIC] program, and the District Council approved it despite their concerns. Now the DHMT is directing the sub-district to carry out these activities but has not set up a team to help coordinate implementation.

- What will sub-district health teams do now?
- What will happen when the local citizen health board [INSERT LOCAL NAME OF ACCOUNTABILTY STRUCTURE] finds out about this?

This year when the central MOH reviewed Xanadu district’s monitoring data for the new program, they noticed that Xanadu had not met any of the targets set by central MOH.

- What would the central MOH do in this situation?
- What would the DHMT do in response?
- Would the District government or the local citizen health board [INSERT LOCAL NAME OF ACCOUNTABILTY STRUCTURE] have anything to say here? Why/why not?
- Have there been any examples like this in your district that you could tell me about? How did that work out?
- How could a situation like this be improved or avoided? Would these be easy or hard things to do?

1. **In general, how much flexibility do you have to get things done/change the way things are done at the district and facility levels?**
2. Why/why not?
3. How much discretion do you have in respect to health budgeting? e.g. funds that are earmarked vs. funds that can be allocated locally.
4. How much discretion does the district health team have over managing health personnel in the district (e.g. hiring, firing, transfers)?
5. How does your level of discretion for planning or budgeting or workforce management affect how you can deliver on your district’s plans and priorities?
6. If you had more flexibility, how would you do things differently?
7. **Is there anything else related to district planning and implementation that we haven’t discussed?**

THANK YOU FOR YOUR TIME.
